# Compartmentalized Neuronal Culture for Viral Transport Research

**DOI:** 10.3389/fmicb.2020.01470

**Published:** 2020-07-15

**Authors:** Yimin Wang, Shan Wang, Hongxia Wu, Xinxin Liu, Jinyou Ma, Muhammad Akram Khan, Aayesha Riaz, Lei Wang, Hua-ji Qiu, Yuan Sun

**Affiliations:** ^1^College of Animal Science and Veterinary Medicine, Henan Institute of Science and Technology, Xinxiang, China; ^2^State Key Laboratory of Veterinary Biotechnology, Harbin Veterinary Research Institute, Chinese Academy of Agricultural Sciences, Harbin, China; ^3^Department of Veterinary Pathology, Faculty of Veterinary and Animal Science, PMAS-Arid Agriculture University, Rawalpindi, Pakistan; ^4^Department of Parasitology and Microbiology, Faculty of Veterinary and Animal Science, PMAS-Arid Agriculture University, Rawalpindi, Pakistan

**Keywords:** dorsal root ganglia (DRG) neurons, compartmentalized neuronal culture, campenot cell culture chambers, microfluidic neuronal culture, alpha herpesvirus, neuronal transport

## Abstract

Neuron-invading viruses usually enter via the peripheral organs/tissues of their mammalian hosts and are transported to the neurons. Virus trafficking is critical for transport or spread within the nervous system. Primary culture of neurons is a valuable and indispensable method for neurobiological research, allowing researchers to investigate basic mechanisms of diverse neuronal functions as well as retrograde and anterograde virus transport in neuronal axons. Primary ganglion sensory neurons from mice can be cultured in a compartmentalized culture device, which allows spatial fluidic separation of cell bodies and distal axons. These neurons serve as an important model for investigating the transport of viruses between the neuronal soma and distal axons. Alphaherpesviruses are fascinating and important human and animal pathogens, they replicate and establish lifelong latent infection in the peripheral nervous system, the mechanism of the viral transport along the axon is the key to understand the virus spread in the nervous system. In this review, we briefly introduce and evaluate the most frequently used compartmentalization tools in viral transport research, with particular emphasis on alphaherpesviruses.

## Introduction

Studying viruses that infect the nervous system remains a challenge, especially determining how viruses spread within the nervous system. Most viruses begin infecting the nervous system from periphery tissue, often at epithelial or endothelial cell surfaces, invading the peripheral nervous system (PNS) or the central nervous system (CNS) along the axon of the neuron. The CNS is well-protected by complex multilayer barriers and immune responses. After virus infection, viruses invade the CNS via two main circuits: the blood supply and peripheral nerves (Swanson and McGavern, 2015). Neurons have long axons, and transport along the axon is necessary for the spread of some viruses (Koyuncu et al., 2013b; Taylor and Enquist, 2015; MacGibeny et al., 2018); therefore, studying the mechanism of virus transport along axons is useful for understanding virus spread in the nervous system.

Isolated primary neurons such as dorsal root ganglia (DRG) neurons and superior cervical ganglia (SCG) (Howard et al., 2014) are widely used for experimental investigation of relevant questions in neurobiology and neurodevelopment and have served as an ideal model for understanding mechanisms of virus spread in the PNS (Tongtako et al., 2017). DRG including the neuronal cell bodies of sensory neurons and glial cells and are located in close proximity to the dorsal root of the spinal cord. DRG neurons are first-order neurons essential for the afferent transport of sensory information through long axons from the limbs and trunk into the CNS. DRG have pseudo-unipolarized morphology with well-defined cellular compartments, extremely long axons, and soma (Belmonte and Viana, 2008; Haberberger et al., 2019). The differentiation of sensory neurons from induced pluripotent stem cells (IPSCs) or embryonic stem cells (ESCs) which share the same properties with DRG neurons and also become ideal cell type to investigate the virus spread in the neurons (Kitazawa and Shimizu, 2011; Grigoryan et al., 2012; Lee et al., 2012; Rowe and Daley, 2019; Umehara et al., 2020). Conventional neuron culture methods do not allow manipulation and control of the culture environment between the soma and axon with enough spatial and temporal resolution to explore virus infection. Several new methods and techniques, consisting of compartmentalized neuronal culture tools, have therefore been developed for exploring virus spread in neurons through the axon. We summarize currently used methods in the field of compartmentalized platforms for understanding viral infection and transport in the nervous system.

## Alphaherpesviruses

Alphaherpesviruses (αHV) belong the family *Herpesviridae* comprising 41 species. Most are neurotropic viruses [such as herpes simplex virus (HSV), pseudorabies virus (PRV), and varicella-zoster virus (VZV)] and invade the nervous system when infecting their natural host. Herpesviruses infect epithelial cells, resulting in invasion of the PNS. However, the mechanism of invasion and transport of αHV to neurons remains elusive.

HSV and VZV are prevalent and important human pathogens with seroprevalence of ~80% in adults (Thaljeh et al., 2019). HSV-1 causes cold sores; it replicates in mucosal epithelia and is transmitted to nerve endings. Transmission is retrograde along the axon to the peripheral ganglia (Stults and Smith, 2019), where the virus can establish long-lasting latent infection; Reactivation of HSV can be triggered by a variety of internal or external stimuli to result in the production of infectious virus, and release of it to the mucosal epithelia by anterograde transport (Wilson and Mohr, 2012). VZV, causative agent of chickenpox, can also establish a latent infection in the nervous system (Mahalingam et al., 2019). Its reactivation in a dorsal root ganglion can lead to herpes zoster with debilitating chronic pain (Laemmle et al., 2019).

PRV is not a human pathogen, but its genome structure and function are similar to those of VZV and HSV (Szpara et al., 2011). Its natural hosts are pigs, but it can infect a broad range of animals. PRV is easy to culture in the laboratory, with rodent and chicken embryo models widely used to study mechanisms of PRV spread and replication in neurons (Pomeranz et al., 2005). All these features make PRV a perfect model for herpesvirus research. Bidirectional transport along the axon enables PRV invasion, replication, and spread in PNS neurons (Koyuncu et al., 2013a).

**Figure 1:**
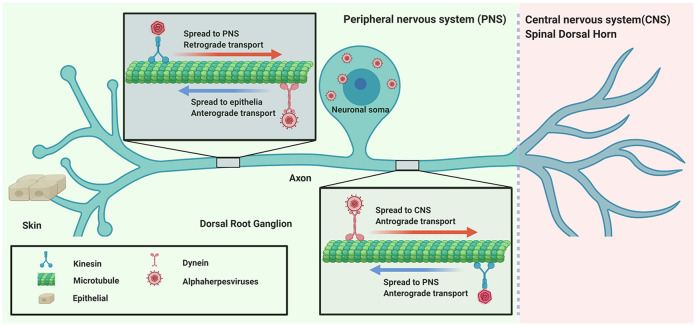
Directional spread of neuroinvasion viruses (alphaherpesviruses) entering the nervous system.

αHV usually infect the mucosal surface of the host and then invade the nervous system by retrograde transport. Subsequently, the virus spreads to nerve endings to egress or enter the CNS by anterograde transport (Figure 1).

Understanding how viruses are transmitted from the mucosal surface to the nervous system can help avoid viral infection of neurons. It is therefore critical and necessary to understand the actively transport and spread of viruses in the neurons. By learning how viruses infect the nervous system and travel in neurons, we can mimic this process to study and treat related neurological diseases.

## Compartmentalized Neuronal Culture

The most frequently used compartmentalized neuronal culture systems are the Campenot chamber and microfluidic cell culture. These tools enable manipulation and separation of the soma and axons.

### Campenot Chamber

In 1977, the compartmentalized culture system known as the “Campenot chamber” was introduced by Campenot (1977). This three-chamber compartmentalized culture system was used to investigate nerve growth factor (NGF) control over the outgrowth of neurites from sympathetic neurons *in vitro*. It allows neurons to be seeded in one chamber and neurites to extend to other chambers, resulting in spatial separation of the neurons and axon. The fluidic separation of the chamber meets the requirements for NGF treatment of cell bodies or axons separately.

**Figure 2:**
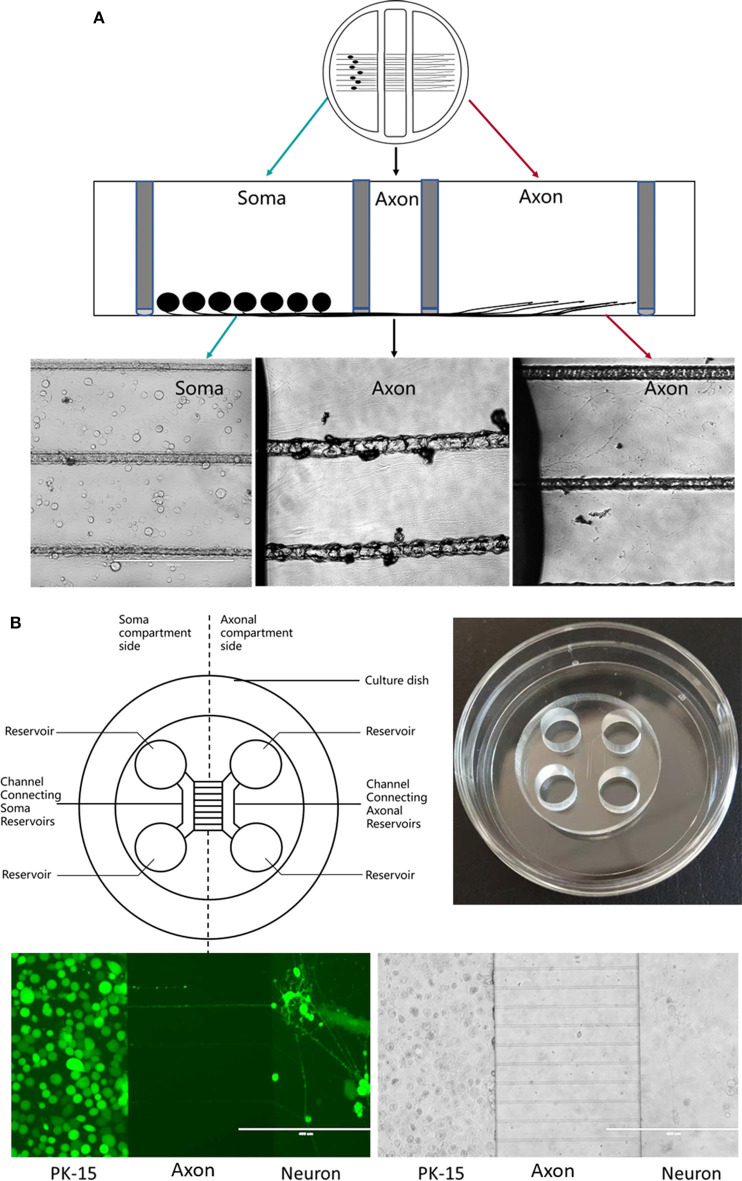
**(A)** Schematic diagram of a Campenot chamber. The neurons are placed in one chamber and extend their axons into the distal chamber. **(B)** Schematic diagram of microfluidic device.

Briefly, silicone grease is used to seal the chamber to a tissue culture dish pre-coated with collagen and scratched to form parallel tracks (Figure 2A). This separates the three chambers while allowing axonal growth underneath the watertight silicone grease into a separate compartment (Pazyra-Murphy and Segal, 2008; Campenot et al., 2009). This method has been extensively used to study various neuronal functions, permitting analysis of RNA, protein, and lipids in both neuronal soma and distal axons separately during neuronal culture (Campenot, 1982, 1994; Karten et al., 2005). This has helped in studying neurotrophin signaling in the transport in the neurons (Wojaczynski et al., 2015; MacGibeny et al., 2018). Campenot chambers have been used by the Enquest laboratory to investigate viral transport (Kramer et al., 2012; Smith, 2012; Kratchmarov et al., 2013). The unique design of the Teflon ring makes it easy to infect the neurons distally or proximally. In this tri-chamber system, neuronal soma and their long axons are separately maintained in different chambers, allowing establishment of fluidic milieu isolated over each segment of the neuron, fluids in three chambers are separated and do not mix. Neuroinvasion viruses can therefore be inoculated into either the chamber containing the neuronal soma or the chamber with distal axons. If infection begins at the neuronal soma, viral particles will be transmitted anterogradely to distal axons along microtubules; in contrast, viral particles may display retrograde transport along microtubules to the soma from distal axons (Smith, 2012; Koyuncu et al., 2013b, 2015; Scherer et al., 2016; Song et al., 2016) (Figure 2A).

### Compartmentalized Microfluidic Culture

Compartmentalized microfluidic technology has provided advanced platforms for neuroscience research over several decades (Zanto et al., 2011). It combines microfluidic, microfabrication, and surface micropatterning techniques to generate a neuronal culture system in a multicompartment device (Park et al., 2006). Application of microfluidics in the field of cell biology is becoming a powerful tool, due to its ability to precisely control, manipulate, and monitor sub-millimeter volume cellular culture environments (Taylor et al., 2005; Whitesides, 2006). Initially, microfluidic devices were designed for chemical and biomolecular analyses with higher sensitivity than traditional methods (Atencia and Beebe, 2005; Kim et al., 2018); more recently, they have been applied in cell biology (Dexter and Parker, 2009; Lo and Yao, 2015; Zhao et al., 2019).

Polydimethylsiloxane (PDMS) is the most popular material for fabricating microfluidic chambers using soft lithography techniques, with a plasma or non-plasma bonding method (Harris et al., 2007) used to seal the chamber to a glass substrate (Figure 2B). The chambers are divided by a barrier into which micron-size grooves are engraved (Taylor and Jeon, 2010), allowing neurites past the chambers (Park et al., 2006; Van Laar et al., 2019).

### Advantages and Disadvantages of Campenot Chambers and Compartmentalized Microfluidic Culture Systems

Compartmentalized culture is becoming a valuable and powerful tool for neuroscience studies (Gross et al., 2007; Coluccio et al., 2019). It is difficult to manipulate and control the axonal growth of neurons in tissue culture plates with isotropic environment because establishment of neuronal polarity involves spontaneous and randomly orientated outgrowth. Compartmentalized culture systems confer a major advantage over single-chamber cell cultures by spatially separating the cellular compartment, the soma, and extension of the axon (Atencia and Beebe, 2005; Fantuzzo et al., 2019).

The Campenot chamber culture method has several disadvantages: (1) The device is difficult to assemble for the early users; (2) mechanical disturbance of the chamber may lead to leak owing to the silicon grease and disrupt the growth of neurites; (3) it is difficult to adapt the technique for sophisticated microscopy, owing to the structure of inner barrier and un-optical material, such as confocal microscopy and live cell imaging during the viral transport along the axon.

Microfluidics utilizes PDMS, which is easy to fabricate, low cost, and has optical properties compatible with high-resolution microscopy, including confocal microcopy (Chiu et al., 2017). It is also non-toxic and permeable to gas, providing alternatively solution for compartmentalized neuronal cell culture (Park et al., 2006; Neto et al., 2016). it offers optical transparency, biocompatibility, and spatial and temporal control for manipulating cellular microenvironments.

The disadvantages of microfluidic devices are as following: (1) The PDMS surface can absorb small hydrophobic molecules (Wang et al., 2012); (2) limited number of axons entering the microfluidic grooves; (3) small volumes of media and constant requirements for fluidic flow to maintain diffusion of small molecules; (4) frequency of channel “blockage” during construction and reuse.

Most of these disadvantages of two methods can be surmounted by skilled operation or redesign the model for fabricating the microfluidic device (Park et al., 2009). All these properties make Campenot chamber and microfluidic culture are ideal and best tool for neuron growth and transport research.

There are still some other compartmentalized devices which are modified based on the “Campenot chamber” or microfluidics, such as two chambers device (De Regge et al., 2006; Hafezi et al., 2012) and redesigned microfluidic culture device (Park et al., 2009).

## Application of Compartmentalized Devices in Neuronal Transport Models *in vitro*

Neuroinvasive viruses spread by infecting nerve terminals and transporting to the soma or other neurons, and by egressing from the soma to the nerve terminals utilizing cellular anterograde and retrograde transport.

Neurons are polarized cells with very long axons, which generally arise from the cell soma and synaptic contacts with other neurons or neurites. Axonal transport is an essential cellular process responsible for trafficking cargo such as mRNA granules, proteins, lipids, mitochondria, synaptic vesicles, autophagosomes, lysosomes, and organelles to their destinations along a polarized microtubule network using kinesin and dynein motor proteins (Terenzio et al., 2017; Sleigh et al., 2019). Anterograde transport is movement of newly biosynthesized proteins and lipids, and organelles from the soma to the synapse or cell membrane of distal axons utilizing the motor protein kinesin (Hirokawa and Tanaka, 2015). In the opposite direction, retrograde transport is involved in maintaining homeostasis by shuttling organelles and aging proteins from axon termini to the cell soma for degradation and recycling of components (Maday, 2016; Ferguson, 2018) utilizing the cytoplasmic protein dynein (Reck-Peterson et al., 2018).

Several studies have identified viruses that are transported along the axon. When αHV enters into axons after membrane fusion, the capsid tegument protein VP1/2 recruits dynein motor proteins for retrograde transport to the cell soma (Zaichick et al., 2013); however, kinesin motor proteins can also bind purified capsid tegument complexes, suggesting that VP1/2 participates in multiple intracellular axonal transport processes during virus replication (Smith, 2012). During αHV egress and spread, the viral membrane glycoprotein gE/gI and US9 protein of PRV and HSV participate in anterograde transport to direct progeny virions either into peripheral tissues or into the CNS (Smith, 2012; Kratchmarov et al., 2013). Interactions between viral proteins and cellular factors are complex, requiring further study using these techniques.

## Conclusions and Future Prospects

In conclusion, compartmentalized culture methods can be used to investigate viral transport. The most common and popular are the “Campenot chamber” and microfluidic culture system, and these methods provide the broadest picture of viral transport mechanisms.

Compartmentalized neuronal culture devices overcome the restrictions of conventional culture methods, making it easy and possible to understand the roles of viral proteins and neuronal proteins in axonal transport. This method can also be applied to co-culture epithelial or glial cells to study the role of these cells during the transport of the virus along the axon. By understanding how viruses move in axons, we can obtain more insight into the process of viral transmission and take measures to control it, especially the threat of latency of herpesviruses.

However, many questions still need to be addressed, such as how transport is coordinated by viral and cellular factors; how virion assembly affects the direction of transport; and how viral proteins and motor proteins are assembled.

## Author Contributions

YW and YS designed the concept of the review article. YW, SW, HW, and YS wrote the manuscript. XL, JM, LW, HQ, MK, and AR helped with revision of the manuscript. All authors read and approved the final manuscript.

## Conflict of Interest

The authors declare that the research was conducted in the absence of any commercial or financial relationships that could be construed as a potential conflict of interest.
